# Nutritional Profile and Functional Properties of Orange‐Fleshed Sweet Potato, Bambara Groundnut, and Brown Rice Blended Complementary Food

**DOI:** 10.1155/ijfo/5397972

**Published:** 2025-10-14

**Authors:** Doreen Ehornam Alomatu, Shadrach Yankey, Linda Gyimah, Guy Eshun

**Affiliations:** ^1^ Department of Food and Nutrition Education, University of Education, Winneba, Ghana, ue.edu.pk; ^2^ Department of Food Science and Technology, Kwame Nkrumah University of Science and Technology, Kumasi, Ghana, knust.edu.gh

**Keywords:** Bambara groundnut, brown rice, food and nutrient security, orange-fleshed sweet potato, vitamin A deficiency

## Abstract

A sweet potato–based complementary food was formulated to enhance the use of locally available ingredients to address micronutrient deficiencies, particularly vitamin A deficiency in Ghana. Five complementary food blends were formulated using orange‐fleshed sweet potato (OFSP), Bambara groundnut, and brown rice flours with the possibility to meet the nutritional requirements of infants aged 6–23 months in alignment with the CAC standards. A sensory evaluation was conducted, consisting of breastfeeding and nonbreastfeeding mothers, to identify the most suitable blend, after which the nutritional profile and functional properties of the preferred blend were analyzed. The most preferred formulation was Formula 5 (60% OFSP, 30% Bambara groundnut, and 10% brown rice flours). It exhibited significantly (*p* < 0.05) higher levels of protein (14.24%), fiber (4.50%), ash (2.77%), carbohydrate (73.78%), and *β*‐carotene (541.4 mg/100 g) compared to the commercial complementary food (CCF), which contained 13.27%, 1.33%, 1.65%, 62.88%, and 0.21 mg/100 g, respectively. In addition, it had a higher concentration of calcium (185.3 mg/100 g), iron (5.84 mg/100 g), potassium (261.33 mg/100 g), and magnesium (204.75 mg/100 g) than the CCF (1.05, 0.99, 38.67, and 179.41 mg/100 g, respectively). The blend exhibited optimal functionality, including a water absorption capacity (WAC) of 140.95%, a swelling power of 9.25 g/g, a solubility index of 14.01%, and a bulk density of 0.64 g/mL. The formulated sweet potato–based complementary food demonstrated a favorable nutritional and functional profile that supports the basic dietary needs of infants aged 6–23 months. It provides adequate levels of protein, carbohydrates, dietary fiber, essential minerals, and *β*‐carotene, a precursor of vitamin A. These attributes align with the CAC standards for infant nutrition and have the potential to help address common micronutrient deficiencies, particularly vitamin A deficiency.

## 1. Introduction

According to WHO recommendations, after 6 months of exclusive breastfeeding, infants should transition to semisolid foods as part of the weaning process. This phase, known as complementary feeding, is crucial for meeting the infant′s increasing nutritional demands, since from this stage, breast milk alone no longer provides all the necessary nutrients [1]. The introduction of nutritionally adequate complementary foods is essential during this period, as improper choices can lead to deficiencies in vital macronutrients and micronutrients, negatively impacting growth and development [1]. Ensuring that complementary foods are nutrient‐dense and appropriate for infants is critical for their overall health and development.

In Ghana, various complementary foods have been developed using a range of locally sourced ingredients, primarily focusing on addressing widespread nutritional deficiencies, such as iron (Fe), protein, and vitamin A deficiencies among children [2]. These efforts have been crucial in combating malnutrition and promoting child health [3]. However, to achieve the most significant impact, these complementary foods must be specifically tailored to meet the unique nutritional needs of infants and young children [4].

Orange‐fleshed sweet potato (OFSP) has emerged as a promising ingredient for creating tailored complementary foods with enhanced nutritional profiles necessary to meet the dietary needs of infants in Ghana [5]. This is largely attributed to its elevated *β*‐carotene content, a crucial precursor to vitamin A, which plays a vital role in promoting eye health and overall immune function [6]. This initiative has increased consumption and diversified the application of OFSP, leading to greater acceptance of the crop among various consumer groups, particularly children [5]. By incorporating OFSP into complementary foods, the naturally high *β*‐carotene content can be leveraged to improve the nutritional quality of meals aimed at reducing vitamin A deficiency [5].

However, despite the high *β*‐carotene content of OFSP, its relatively low protein and fat contents highlight the need for supplementation with other nutrient‐dense ingredients [5]. To create a well‐rounded complementary food with superior nutritional qualities, it is essential to incorporate legume and cereal ingredients that are rich in protein and essential fats [7]. Legumes such as Bambara groundnut and cereals like brown rice are excellent candidates, as they can significantly enhance the overall nutritional profile by providing the necessary macronutrients for infant growth and development [8]. Bambara groundnut (*Vigna subterranea*) is increasingly recognized for its superior qualities as a complementary food ingredient, particularly in addressing nutritional deficiencies in developing countries [9]. Its high‐protein content, healthy fats, essential amino acids, and beneficial micronutrients make it an excellent choice for enhancing dietary quality [10]. Its protein content is comparable to or even superior to that of other legumes, making Bambara groundnut an excellent source of protein for children, who require adequate protein for growth and development [11]. The protein in Bambara groundnut is also rich in essential amino acids, particularly lysine, which is often limited in cereal‐based diets. This characteristic is crucial for improving the overall protein quality of complementary foods, especially when combined with cereals, which typically lack sufficient lysine [11].

The strategy of blending different ingredients would ensure that the OFSP complementary food not only addresses vitamin A deficiency but also delivers adequate protein, healthy fats, and other essential nutrients, meeting the dietary requirements for optimal infant nutrition. Therefore, the aim of this study is to develop a complementary food using OFSP (*Beauregard* variety), Bambara groundnut, and brown rice. The research further investigates the characteristics of the formulated complementary food, focusing on its sensory acceptability, nutrient composition, and functional and pasting properties. A preprint version of this manuscript has previously been published [12].

## 2. Materials and Methods

### 2.1. Source of Raw Materials

The *Beauregard* variety of OFSP was obtained from the farms of the Crops Research Institute (CSIR‐CRI), Fumesua. Kumasi brown rice was purchased from the farms of local rice growers, Adansi Praso. The cream with black eye variety of the Bambara groundnut was purchased from the local market in Jacobu.

### 2.2. Preparation of OFSP, Brown Rice, and Bambara Groundnut Flours

For OFSP, the method of Marcel et al. [13] was used with some modification. Freshly harvested OFSP was sorted, washed with potable water, peeled with a stainless steel knife, grated into 0.5 cm pieces, and then solar‐dried for 48 h until the moisture content dropped below 10%. The dried OFSP chips were then milled using a locally made hammer mill and sifted through a 210 *μ*m sieve to obtain the flour. Brown rice was winnowed, sorted, washed in portable water, and dried according to the protocol of Marcel et al. [13]. The dried brown rice was then milled and sifted into flour. Following the modified method of Alawode et al. [14], Bambara groundnuts were first sorted and soaked in water for 12 h. They were then dehulled and dried using a locally fabricated solar drier for 216 h until a final moisture content of about 11.0% was achieved. This ensured uninterrupted drying throughout the period. After drying, the groundnuts were roasted over low heat for 15 min. The roasted groundnuts were subsequently milled and sifted through a 210 *μ*m sieve to obtain the flour.

### 2.3. Proximate and Mineral Analysis of Samples

The proximate composition of the raw materials, flour samples, and the formulated product was analyzed using standard AOAC methods [15]. Specifically, moisture content was determined by oven‐drying at 105°C (AOAC 925.10), protein by the Kjeldahl method (AOAC 979.09), fat using Soxhlet extraction with petroleum ether (AOAC 920.39), ash by incineration in a muffle furnace at 550°C (AOAC 923.03), and fiber by acid and alkaline digestion (AOAC 962.09). Carbohydrate was calculated by difference.

Mineral content (Fe, zinc [Zn], phosphorus [P], potassium [K], calcium [Ca], and magnesium [Mg]) was determined following the modified procedure of Laryea et al. [5]. One gram of each sample was weighed into a digestion tube, and 15 mL of concentrated nitric acid (HNO_3_) was added. Samples were digested for 30 min at 150°C in a fume chamber. After cooling, 10 mL of perchloric acid (70% HClO_4_) was added, and digestion continued at 200°C until a clear solution was obtained. The digested solution was allowed to cool, diluted with 80 mL of distilled water, boiled for 10 min, and filtered through Whatman No. 42 filter paper into a 250‐mL volumetric flask. The volume was made up to the mark with distilled water. The concentrations of minerals were determined using atomic absorption spectrophotometry (Analytik Jena novAA 400P, Germany) for Fe, Zn, Ca, Mg, and P. K was measured using a flame photometer (Jenway Digital, Model PFP7, United States).

### 2.4. Formulation of Complementary Food

Based on the macronutrients (carbohydrate, protein, and fat) of the individual flours, material balance equations were applied to estimate the minimum amount of each ingredient required to meet the recommended macronutrient composition for complementary foods as specified by the CAC [16]. Based on this analysis, upper and lower bounds (ranges) for each ingredient′s proportion in the blend were determined. These ranges were then inputted into Design‐Expert software (Version 13, Stat‐Ease Inc., United States) to generate five optimized complementary food blends using a D‐optimal mixture design. The final formulations obtained are presented in Table 1.

**Table 1:** Formulations for the complementary food blend.

**Code**	**Flour compositions (%)**			**Other ingredients (g/100 g of flour sample)**	
**Formula**	**OFSP**	**Bambara groundnut**	**Brown rice**	**Sugar**	**Milk**
1	65	25	10	5	5
2	55	35	10	5	5
3	55	30	15	5	5
4	62	25	13	5	5
5	60	30	10	5	5

### 2.5. Sensory Evaluation

#### 2.5.1. Preparation of Samples

##### 2.5.1.1. Commercial Complementary Food (CCF) (Control Sample) and Blends

This study used a CCF as the control sample, and it was included in the sensory evaluation. To prepare the samples, 250 mL of water was brought to a boil in a cooking utensil. A 100 g portion of both the complementary food blend and the control sample was mixed with 250 mL of water to form a slurry, which was then added to the boiling water. The mixture was stirred continuously with a wooden ladle. For the OFSP complementary food, 5 g of sugar and 5 g of milk powder were added, and the mixture was cooked for 15 min until a soft paste was achieved.

##### 2.5.1.2. Sensory Evaluation

The sensory evaluation took place in a dedicated sensory laboratory within the Department of Food Science and Technology at Kwame Nkrumah University of Science and Technology (KNUST), in a closed room with sufficient natural lighting. Panelists were seated in individual booths for the evaluation, which was conducted over 2 days between 10:00 a.m. and 3:00 p.m. Forty untrained panelists, consisting of 28 breastfeeding mothers and 12 nonbreastfeeding mothers with prior experience in feeding babies, were asked to assess coded samples of complementary foods. They evaluated attributes such as color, thickness, consistency, sweetness, taste, aftertaste, and overall acceptability using a 5‐point hedonic scale (1 = *dislike very much*, 5 = *like very much*). Water was provided to cleanse the palate between tastings.

##### 2.5.1.3. *β*‐Carotene Determination for Selected Blended Composite Food and OFSP Flour

The *β*‐carotene content of the most preferred complementary food blend, OFSP flour, and a CCF was determined using high‐performance liquid chromatography (HPLC), following the procedure described by Munasinghe and Wansapala [17], with slight modifications. Firstly, a standard *β*‐carotene solution (5 mg/100 mL in n‐hexane) was prepared and serially diluted to 1 *μ*g/mL for calibration. Approximately 1 g of each sample was accurately weighed into a 25‐mL volumetric flask and dissolved with 25 mL n‐hexane. The flask was sealed, shaken, and stored in the dark for 12 h to allow full extraction of carotenoids. The solution was then centrifuged at 500 rpm for 15 min. The organic phase was collected after centrifugation, and the extraction was repeated on the residue with 25 mL n‐hexane, followed by centrifugation at 500 rpm for 15 min. Extractions were continued until the residues turned colorless. The organic phases were combined for HPLC analysis. HPLC analysis was performed using a Shimadzu system equipped with an LG6 pump, UV‐visible detector (*λ* = 450 nm), CR6 recorder, ODS reverse‐phase column (C18), and a Rheodyne 1725 injector. The mobile phase, delivered isocratically, consisted of acetonitrile (70%), dichloromethane (20%), and methanol (10%), delivered at a flow rate of 1.0 mL/min. The column temperature was maintained at 30°C, and 50 *μ*L of each sample was injected for analysis. The *β*‐carotene content of the samples was quantified by comparing the retention times and peak areas with those of the standard, and results were expressed in parts per million (ppm). All analyses were conducted in duplicate.

##### 2.5.1.4. Color Determination

Surface color measurements were determined using a CR‐400 Chroma Meter (Minolta Co., Osaka, Japan), which consisted of a measuring head (CR‐400) with an 8 mm diameter measuring area and two standard observers, and a data processor. The chroma meter was calibrated on the Hunter Lab color space system using a white tile (D65: *Y* = 85.1, *x* = 0.3168, *y* = 0.3236). Hunter *L* (lightness), *a* (redness), and *b* (yellowness) values were measured in duplicate on samples.

##### 2.5.1.5. Pasting Profile Determination

The pasting properties of the flour samples and formulated complementary food were determined using a Rapid Visco Analyzer (RVA 4500, Perten Instrument, Australia), following the method of Effah‐Manu et al. [18] with slight modifications. Approximately 3 g of the sample (on a 14% moisture basis) was weighed and mixed with 25 g of distilled water in an RVA canister to obtain a total suspension weight of 28 g. The sample was thoroughly stirred to ensure a uniform slurry. Samples were heated to and held at 50°C for 1 min, heated to 95°C at 6°C/min, and held for 1 min. This was followed by cooling to 50°C with another 1 min holding time. Corresponding values for peak viscosity, trough viscosity (minimum viscosity at 95°C), final viscosity (viscosity at 50°C), breakdown viscosity, and setback viscosity were read on a computer connected to the RVA.

##### 2.5.1.6. Functional Profile Determination

Water absorption capacity (WAC), swelling power, solubility index, and bulk density were determined using the method described by Effah‐Manu et al. [18] with slight modifications as detailed below.

##### 2.5.1.7. WAC

Approximately 3.0 g of the sample was weighed into a previously weighed centrifuge tube, and 20 mL of distilled water was added. The mixture was vortexed for 2 min and left to stand at room temperature (25°C) for 30 min. After standing, it was centrifuged at 3000 rpm for 30 min. The supernatant was carefully decanted, and the weight of the wet sediment was recorded. The WAC was calculated as follows:

WAC%=weight of wet sedimentdry sample weight×100.

### 2.6. Swelling Power and Solubility Index

One gram (1 g) of the sample was weighed into a 40‐mL capacity centrifuge tube (preweighed), and 40 mL of distilled water was added. The mixture was stirred gently and uniformly to avoid granule rupture and vortexed at low speed for 1 min. The suspension was heated in a thermostatically controlled water bath at 85°C with constant stirring, then cooled to room temperature. It was centrifuged at 2200 rpm for 15 min.

The supernatant was poured into a preweighed crucible and evaporated to dryness in an oven at 105°C. After cooling, the crucible was reweighed, and the weight of the dried supernatant was recorded. The swelling power was calculated using the weight of the sediment obtained after centrifugation:

Solubility index%=weight of dried supernatantinitial sample weight×100,

Swelling power=weight of sedimented pasteinitial sample weight×100.

### 2.7. Bulk Density

Ten grams (10 g) of the sample was weighed into a 50‐mL graduated measuring cylinder. The cylinder was tapped repeatedly on a flat surface until no further volume reduction was observed. The volume of the packed sample was recorded, and the bulk density was calculated as follows:

Bulk density g/mL=weight of samplegvolume of packed sample mL.

### 2.8. Statistical Analysis

All data, except for the sensory evaluation, were collected in triplicate. The results were expressed as means and standard deviations. Statistical analysis was conducted using one‐way analysis of variance (ANOVA), followed by Tukey′s HSD test to identify significant differences at a 95% confidence level.

## 3. Results and Discussion

### 3.1. Proximate and Mineral Composition of OFSP, Bambara Groundnut, and Brown Rice Flour

The flour yield after drying and milling 100 g of the samples was highest for brown rice (96.6%), followed by Bambara groundnut (83.4%) and OFSP (27.3%).

The proximate composition analysis revealed distinct nutritional profiles for brown rice flour, Bambara groundnut flour, and OFSP flour, reflecting their potential applications in food product development. Brown rice flour exhibited the highest carbohydrate content (85.25%), followed by Bambara groundnut flour (69.38%) and OFSP flour (69.09%) (Table 2). The elevated carbohydrate content in brown rice flour aligns with its role as a primary energy source, making it a suitable base for energy‐dense foods. However, the carbohydrate content of OFSP flour in this study was notably lower than the 87.09% reported by Nchung et al. [19]. This discrepancy may stem from varietal differences, as OFSP cultivars vary in starch accumulation due to genetic factors, environmental conditions, or postharvest processing methods. For instance, higher starch content in certain OFSP varieties could explain the elevated carbohydrate levels in Laryea et al.′s study. These findings suggest that careful selection of OFSP varieties is critical when targeting specific nutritional outcomes in food formulations.

**Table 2:** Proximate composition of Bambara groundnut, brown rice, and sweet potato flour.

**Variable**	**BGF**	**BRF**	**OFSPF**
Carbohydrate (%)	68.63 ± 0.03^a^	80.54 ± 0.12^b^	84.02 ± 0.12^c^
Fiber (%)	0.29 ± 0.01^a^	0.23 ± 0.01^b^	1.80 ± 0.25^c^
Fat (%)	5.12 ± 0.17^a^	4.96 ± 0.72^b^	2.23 ± 0.07^c^
Protein (%)	22.18 ± 0.67^a^	8.34 ± 0.02^b^	3.27 ± 0.22^c^
Ash (%)	3.03 ± 0.07^a^	1.22 ± 0.01^b^	3.47 ± 0.07^c^
Moisture (%)	0.75 ± 0.15^a^	4.71 ± 0.58^b^	5.21 ± 0.12^c^
*β*‐Carotene (mg/100 g)	—	—	457.80 ± 0.00
Iron (mg/100 g)	21.90 ± 0.03^a^	18.20 ± 0.06^b^	70.80 ± 0.05^c^
Zinc (mg/100 g)	5.00 ± 0.69^a^	1.40 ± 0.04^b^	8.00 ± 0.70^c^
Phosphorus (mg/100 g)	0.37 ± 0.01^a^	0.13 ± 0.01^b^	0.21 ± 0.01^c^
Potassium (mg/100 g)	1.93 ± 0.05^a^	0.99 ± 0.06^b^	2.62 ± 0.07^c^
Calcium (mg/100 g)	0.36 ± 0.02^a^	0.28 ± 0.01^b^	0.48 ± 0.04^c^
Magnesium (mg/100 g)	0.15 ± 0.04^a^	0.12 ± 0.01^b^	0.25 ± 0.01^c^

*Note:* Values are mean ± SD (*n* = 3). Mean values with different superscripts (a, b, and c) along the row are significantly different at a 95% confidence level.

Abbreviations: BGF, Bambara groundnut flour; BRF, brown rice flour; OFSPF, orange‐fleshed sweet potato flour.

In terms of dietary fiber, OFSP flour had the highest content (21.94%), significantly surpassing Bambara groundnut flour (0.29%) and brown rice flour (0.23%). This high fiber content in OFSP flour positions it as a valuable ingredient for developing functional foods aimed at improving digestive health or managing blood sugar levels. However, the fiber content observed here was substantially higher than the 3.5% reported by Sebben et al. [20] and the 6.13%–7.11% range reported by Etana et al. [21]. These differences could be attributed to variations in analytical methods in the fiber extraction or differences in OFSP processing, which can affect fiber retention. The low fiber content in brown rice and Bambara groundnut flours suggests they may be less effective for fiber‐enriched products, but could complement OFSP flour in composite flours to balance nutritional profiles.

Bambara groundnut flour, as a legume, was the richest source of protein (22.18%) and fat (5.12%), followed by brown rice flour (8.34% protein and 4.96% fat), with OFSP flour having the lowest levels (3.27% protein and 2.23% fat). The high‐protein content of Bambara groundnut flour underscores its potential as a plant‐based protein source, particularly in regions where animal proteins are scarce or costly. Compared to the 19.0% protein reported by Linnemann [22], the higher protein content in this study may reflect the use of a high‐protein Bambara groundnut variety. Similarly, the fat content aligns with the 5.3%–7.8% range reported by Hillocks et al. [23], but it was lower than that found by Mazahib et al. [24]. These variations likely arise from differences in Bambara groundnut cultivars, soil nutrient profiles, or extraction methods, which can influence lipid accumulation. The relatively low protein and fat content in OFSP flour, while limiting its use as a primary protein source, enhance its suitability for low‐fat, carbohydrate‐rich products.

OFSP flour exhibited the highest ash content (3.47%), indicating a richer mineral load compared to Bambara groundnut flour (3.03%) and brown rice flour (1.22%). Ash content serves as an indirect estimate of the total mineral content in food, and its variation among the three flours can be attributed to differences in their physiological roles and environmental adaptations. Sweet potatoes, being root tubers, accumulate more minerals such as Fe, Zn, K, Ca, and Mg from the soil. This is evident from their respective concentrations, with OFSP flour containing the highest levels of Fe (70.80 mg/100 g), Zn (8.00 mg/100 g), K (2.62 mg/100 g), Ca (0.48 mg/100 g), and Mg (0.25 mg/100 g). In contrast, Bambara groundnut, a leguminous seed, demonstrated intermediate mineral levels (Fe [21.90 mg/100 g], Zn [5.00 mg/100 g], K [1.93 mg/100 g], Ca [0.36 mg/100 g], and Mg [0.15 mg/100 g]), but had the highest P content (0.37 mg/100 g). This is consistent with the fact that legumes typically store more P in the form of phytic acid in seeds to support germination. Brown rice flour, being a cereal grain, exhibited the lowest ash and mineral content, which aligns with prior findings showing cereals generally have lower mineral densities, partly due to the milling process and their lower uptake efficiency of certain micronutrients compared to tubers and legumes [25]. Differences in mineral composition may also result from agronomic factors such as soil type, pH, fertilizer application, and varietal genetics. For example, the high Fe and Zn levels in OFSP flour might be related to biofortified varieties or cultivation in mineral‐rich soils [25]. The variation in ash content across studies, such as the higher ash values in this study compared to the 0.5%–0.09% range reported by Wireko‐Manu and Amamoo [26] and Honi et al. [27], could therefore stem from varietal differences and cultivation conditions. The complementary contribution of these three flours, each rich in different minerals, enhances the nutritional balance of the developed food product. OFSP improves the Fe, Zn, and Ca profile, whereas Bambara groundnut adds significant P and protein, while brown rice contributes to carbohydrate density and some essential micronutrients. Their synergistic use is beneficial in formulating nutrient‐dense complementary foods to address micronutrient deficiencies common in vulnerable populations.

The *β*‐carotene content of OFSP flour was 457.80 mg/100 g, confirming its potential as a rich source of provitamin A. This high *β*‐carotene level is consistent with the OFSP used, which is known for its elevated carotenoid content. The moisture content was highest in OFSP flour (5.21%) compared to Bambara groundnut and brown rice flours. This higher moisture level may be attributed to the relatively higher fiber and carbohydrate content of OFSPs, which enhances water‐holding capacity during drying [28]. Dietary fiber, particularly soluble fiber, forms a gel‐like matrix that retains more moisture, while the starchy structure of OFSP tissues can also trap water molecules. Moreover, the cellular structure of root crops, such as OFSPs, is more porous than that of legumes or cereals, contributing to higher moisture retention even after drying. These compositional and anatomical characteristics likely explain the observed differences in residual moisture content across the flours.

### 3.2. Panelists′ Acceptability of the Complementary Food

The acceptability of the formulated complementary food, based on all tested parameters, was influenced by variations in the composition of the OFSP–Bambara groundnut–brown rice blends (Table 3). The acceptability scores ranged from “neither like nor dislike (3)” to “like moderately (4)” on the 5‐point hedonic scale. Consistency was rated highest for CCF, followed by Formula 4 (62:25:13). Formulas 1 (65:25:10) and 3 (55:30:15) were the least preferred.

**Table 3:** Sensory acceptability of OFSP‐based complementary food.

**Sensory attributes**	**CCF (control)**	**F1 (65:25:10)**	**F2 (55:35:10)**	**F3 (55:30:15)**	**F4 (62:25:13)**	**F5 (60:30:10)**
Consistency	4.15 ± 0.97^a^	3.45 ± 1.24^c^	3.60 ± 1.15^bc^	3.55 ± 1.28^bc^	4.00 ± 1.01^ab^	3.75 ± 1.33^abc^
Aftertaste	4.10 ± 0.63^a^	3.20 ± 1.29^c^	3.58 ± 1.08^bc^	3.38 ± 1.37^bc^	3.80 ± 1.20^ab^	3.85 ± 1.17^ab^
Appearance	4.10 ± 0.93^a^	3.75 ± 1.13^a^	3.85 ± 1.17^a^	4.12 ± 0.82^a^	4.00 ± 1.07^a^	4.12 ± 1.07^a^
Taste	4.18 ± 0.90^a^	3.30 ± 1.29^b^	3.28 ± 1.26^b^	3.55 ± 1.30^b^	3.68 ± 1.29^ab^	3.71 ± 1.38^ab^
Texture	3.00 ± 1.26^a^	4.03 ± 1.12^b^	4.22 ± 1.05^b^	4.08 ± 1.14^b^	4.03 ± 1.31^b^	4.22 ± 1.25^b^
Overall acceptability	4.00 ± 0.68^a^	3.58 ± 1.22^ab^	3.45 ± 1.24^b^	3.55 ± 1.18^ab^	3.35 ± 1.35^b^	3.70 ± 1.18^ab^

*Note:* Sample ratios are represented as orange‐fleshed sweet potato:Bambara groundnut:brown rice. Scale: 1, *dislike very much*; 2, *dislike moderately*; 3, *neither like nor dislike*; 4, *like moderately*; and 5, *like very much*. Mean values with different superscripts along the row are significantly different (*p* < 0.05).

Abbreviation: CCF, commercial complementary food.

The acceptability of aftertaste and appearance was highest for CCF, followed by Formula 5 (60:30:10) and Formula 4 (Table 3). Formula 1 was the least preferred for both aftertaste and appearance. Some panelists noted a slightly bitter aftertaste, which may have influenced the lower ratings of some of the formulas. This bitterness could be attributed to ipomeamarone, a compound in sweet potatoes that develops during dry heat processing. The intensity of the bitter aftertaste is expected to increase with higher proportions of OFSP [5]. In terms of taste, CCF was preferred over Formula 5. For texture, Formulas 5 and 2 (55:35:10) received the highest ratings, followed by CCF, while Formulas 1 and 4 (62:25:13) were the least favored in terms of texture. Regarding overall acceptability, CCF was the most accepted, followed by Formula 5, with Formula 4 receiving the lowest overall acceptability rating.

### 3.3. Nutrient, Color, and Mineral Composition of Complementary Foods

#### 3.3.1. Nutrient Composition

Compared to the CCF sample (Table 4), Formula 5, the most preferred formulation, exhibited significantly (*p* < 0.05) higher levels of protein (14.24%), fiber (4.50%), ash (2.77%), carbohydrate (73.78%), and *β*‐carotene (271.8 mg/100 g). However, Formula 5 ranked second in moisture content (3.16 mg/100 g) and had the lowest fat content (1.56 mg/100 g) when compared to the control sample. The energy content of Formula 5 was 366.08 kcal/100 g, slightly higher than that of OFSP flour, which had 363.65 kcal/100 g but significantly (*p* < 0.05) lower than the CCF value.

**Table 4:** Nutrient and color composition of OFSP‐based complementary food.

	**Complementary food**			
Proximate	F5	OFSPF	CCF	RDI values
Component	(60:30:10)			
Moisture (%)	3.16 ± 0.07^a^	—	3.32 ± 0.20^a^	< 10.0
Protein (%)	14.24 ± 0.16^a^	—	13.27 ± 0.55^b^	5.0–15.0
Fat (%)	1.56 ± 0.13^a^	—	11.02 ± 1.22^b^	12.0–20.0
Fiber (%)	4.50 ± 0.22^a^	—	1.33 ± 1.21^b^	< 5.0
Ash (%)	2.77 ± 0.12^a^	—	1.65 ± 0.12^b^	≥ 5.0
Carbohydrate (%)	73.78 ± 0.12^a^	—	62.88 ± 1.27^b^	40.0–60.0
*β*‐Carotene (mg/100 g)	271.8 ± 0.04^a^	457.80 ± 0.00^b^	0.21 ± 0.22^b^	0.35–0.40
Energy (kcal/100 g)	366.08 ± 1.32^a^	363.65 ± 0.71^a^	—	400.0
Color				
*L*∗	80.24 ± 0.92^a^	85.98 ± 1.13^b^	—	—
*a*∗	4.33 ± 0.19^a^	7.70 ± 0.48^b^	—	—
*b*∗	25.15 ± 0.05^a^	27.37 ± 0.11^b^	—	—

*Note:* Sample ratios are represented as orange‐fleshed sweet potato:Bambara groundnut:brown rice. Mean values with different superscripts along the row are significantly different (*p* < 0.05). Lightness/whiteness or darkness (*L*∗), red to green (*a*∗), yellowness or blueness (*b*∗). RDI values (FAO [29] and CAC/GL 08 [30] guidelines).

Abbreviations: CCF, commercial complementary food; OFSPF, orange‐fleshed sweet potato flour.

According to the FAO [29] and CAC/GL 08 [30] guidelines, the fat content of complementary foods should be between 12.0% and 20.0%. However, the fat content of Formula 5 (1.56%) was lower than the fat levels reported by Laryea et al. [5] (6.20%), Bonsi et al. [31] (4.30%), and Adenuga [32] (2.40%–2.80%). Fat is a crucial macronutrient, providing energy, supporting cell functions, facilitating the absorption of fat‐soluble vitamins, and contributing to cell membrane development [2]. Adequate fat intake is essential for overall health, and insufficient levels can reduce energy density, which may result in inadequate calorie intake, particularly concerning for individuals with high energy needs, such as growing children [2].

According to FAO/WHO/UNICEF [33] and FAO [29], the ash content of complementary foods should not fall below 5.0%. However, the ash content of the selected formula did not meet this standard, as it was below the recommended level. Formula 5 had an ash content of 2.77%, which is within the values reported by Adigwe et al. [34] (2.71%), but lower than that found by Amagloh et al. [2] (2.80%–11.25%). In comparison, Bonsi et al. [31] (1.39%–1.98%) and Haque et al. [35] (1.90%–2.14%) reported lower ash levels in their sweet potato–based complementary foods than those observed in this study. The variations in ash content can be attributed to differences in the raw materials used for sweet potato–based complementary foods. Ash content reflects the mineral composition of the food, including essential minerals. Based on the ash content, Formula 5 appears to offer a suitable mineral composition to meet the nutritional needs of growing infants [23].

The primary source of energy in complementary foods is derived from their carbohydrate content. However, these carbohydrates must be easily digestible for infants and young children [16, 29]. According to FAO [29] and CAC [16] guidelines, the carbohydrate content of complementary foods should fall within the range of 40.0%–60.0% to meet the energy needs essential for infant growth. In this study, the carbohydrate content exceeded the recommended range set by FAO [29] and CAC [16]. It also surpassed the values reported by Adedayo et al. [36] (65.95%) and by Bonsi et al. [31] and Haque et al. [35] (42.30% and 54.5%, respectively). Carbohydrates, along with proteins and fats, are one of the three essential macronutrients that supply energy to the body, particularly supporting brain and muscle function and contributing to various physiological processes. The high carbohydrate content of Formula 5 suggests that it would be capable of providing sufficient energy for the growing infant [22].

According to FAO [29] and CAC [16] guidelines, the protein content of complementary foods should range from 5.0% to 15.0%, with the protein sourced from high‐quality ingredients like legumes, eggs, dairy, meat, fish, and nuts. The protein content in Formula 5 (14.24) met the FAO standard, although it was lower than the values reported by Adenuga [32] (31.50%–38.5%) and higher than the values reported by Nchung et al. [19] (7.37%–14.15%), both of whom also formulated complementary foods using OFSP but with different ingredient combinations. Protein is a vital macronutrient that plays a key role in the growth, development, and maintenance of tissues and organs, especially during infancy, when rapid growth occurs. By meeting FAO standards for protein, Formula 5 ensures that infants receive an adequate amount of this nutrient, which supports healthy weight gain, muscle development, and the formation of essential organs and tissues [37]. Complementary foods should also contain sufficient fiber to aid digestion and bowel function. According to CAC/GL 08 [30] and CAC [16], the fiber content of complementary foods should be below 5.0%, as higher fiber levels can increase bulk and cause flatulence. Formula 5 meets this standard, though its fiber content was higher than the value reported by Adigwe et al. [34] (1.70%). Therefore, Formula 5 can provide adequate fiber to promote healthy digestion in infants, supporting overall digestive health [37].

Vitamin A plays a crucial role in addressing vitamin A deficiency in children, with dietary sources being a key means of providing this nutrient to infants and young children [2]. The recommended daily allowance for infants aged 3 months to 3 years ranges from 0.35 to 0.40 mg of retinol activity equivalents (RAEs) per day, as outlined by Koletzko et al. [38] and Booth et al. [39]. The *β*‐carotene content of Formula 5 was 271.8 mg/100 g, primarily derived from the OFSP (Beauregard variety). Using the standard conversion factor of 24 *μ*g *β*‐carotene = 1 *μ*g retinol equivalent (RE) (FAO/WHO), the *β*‐carotene content of 271.8 mg/100 g corresponds to approximately 11.33 mg RE/100 g.

Thus, 1 g of Formula 5 delivers about 0.1133 mg RE, and for a daily serving of 10–20 g of complementary foods, it would yield 1.133–2.266 mg RE, far exceeding the RDA for infants. Therefore, Formula 5 has the potential to contribute significantly to meeting daily vitamin A requirements and may play a role in alleviating vitamin A deficiency among young children. Additionally, the *β*‐carotene content of Formula 5 was higher than that reported by Laryea et al. [5] (0.53 mg/100 g) for a similar OFSP‐based complementary formula, which could be due to varietal differences and the use of drum‐drying in their study as opposed to the method applied in this work.

#### 3.3.2. Color

The color parameters *L*∗, *a*∗, and *b*∗ (Table 4) showed significantly (*p* < 0.05) higher values for OFSP flour (85.98, 7.70, and 27.37, respectively) compared to Formula 5 (80.24, 4.33, and 25.15, respectively). The higher *L*∗ values indicate greater lightness, meaning that both samples appear brighter, though not necessarily white. The positive *a*∗ values suggest the presence of red‐orange hues, while the *b*∗ values reflect yellowness in the color profile. The combination of elevated *a*∗ and *b*∗ values points to the characteristic orange‐yellow coloration of OFSPs, which is largely attributed to their *β*‐carotene content. These color characteristics enhance both the visual appeal and nutritional perception of the complementary foods, as consumers often associate bright, naturally pigmented colors with higher nutritional value, particularly in provitamin A–rich products.

#### 3.3.3. Mineral Composition

In comparison to the CCF samples (Table 5), Formula 5 exhibited significantly (*p* < 0.05) higher concentrations of Ca at 185.3 mg/100 g, Fe at 5.84 mg/100 g, K at 261.33 mg/100 g, Mg at 204.75 mg/100 g, and Zn at 1.20 mg/100 g.

**Table 5:** Mineral composition of OFSP‐based complementary food.

**Sample**	**Ca (mg/100 g)**	**Fe (mg/100 g)**	**K (mg/100 g)**	**Mg (mg/100 g)**	**Zn (mg/100 g)**
Formula 5 (60:30:10)	185.3 ± 2.60^a^	5.84 ± 0.25^a^	261.33 ± 0.93^a^	204.75 ± 9.75^a^	1.20 ± 0.60^a^
CCF	1.05 ± 0.33^b^	0.99 ± 0.32^b^	38.67 ± 0.13^b^	179.41 ± 5.63^b^	64.66 ± 2.32^b^
RDI (mg/day)	210–500	1.7–11	60–160	30–80	2–3

*Note:* Sample ratios are represented as orange‐fleshed sweet potato:Bambara groundnut:brown rice. Mean values with different superscripts along the column are significantly different (*p* < 0.05).

Abbreviations: CCF, commercial complementary food; RDI, recommended daily intake [38].

The Ca content in the most preferred formulation, Formula 5 (185.3 mg/100 g), was significantly higher than that of the commercial fortified complementary food, CCF (1.05 mg/100 g). Despite being fortified, CCF had much lower Ca levels. Although the Ca content in Formula 5 is slightly below the recommended daily intake (200–260 mg/day), it exceeds the values reported by Adedayo et al. [36]. Enhancing Ca intake could be achieved by adding milk powder, further boosting Ca content to meet the daily requirements.

According to Koletzko et al. [38], the recommended nutrient intake (RNI) for Fe in infants aged 6 months to 3 years ranges from 1.7 to 11 mg/day. Formula 5, with an Fe content of 5.84 mg/100 g, successfully meets this recommended intake, providing an adequate amount of Fe for infant nutrition. Furthermore, the Fe content in Formula 5 is significantly higher than the 0.25–0.37 mg/100 g reported by Adedayo et al. [36] for a similar formulation. This indicates that Formula 5 could be an effective complementary food for addressing Fe needs in young children, contributing to the prevention of Fe deficiency and supporting proper growth and development.

Moreover, the recommended K content in baby formulas for infants typically ranges between 60 and 160 mg/100 g, as established by Koletzko et al. [38]. The K level in Formula 5 exceeded the recommended daily intake, suggesting it provides a more abundant supply of K compared to typical infant formulas. Additionally, Formula 5 had a significantly higher K content than the 20.88–23.28 mg/100 g found in the OFSP‐based complementary food developed by Nchung et al. [19]. The elevated K levels in Formula 5 may enhance its capacity to meet the nutritional needs of growing infants, supporting overall physiological development and well‐being.

Furthermore, the Mg and Zn levels in the most preferred formula aligned with the recommended daily intake for infants, reflecting its adequacy in meeting essential mineral requirements. Although the Mg content in Formula 5 was higher compared to the value reported by Adigwe et al. [34] (127.23–167.72 mg/100 g) and Adedayo et al. [36] (10.65–13.23 mg/100 g), the Zn content fell below the values reported by Nchung et al. [19] (1.85–2.39 mg/100 g) but surpassed Adedayo et al.′s findings (0.53–0.84 mg/100 g), highlighting a notable variation in mineral composition. The higher Zn levels in Formula 5 may contribute to enhanced immune function and growth in infants, while adequate Mg levels support critical biochemical processes, including energy production and muscle function. The observed differences in mineral concentrations across studies could be attributed to several factors, including agricultural practices [40, 41], geographic location, and the specific crop varieties used [42, 43]. Such variables can significantly influence the nutrient profile of raw materials, ultimately impacting the mineral content in complementary foods.

### 3.4. Functional and Pasting Properties of Blended Flour

#### 3.4.1. Pasting Properties

From Table 6, Formula 5 exhibited a pasting temperature of 86.75°C and a peak time of 5.46 min, which were both significantly (*p* < 0.05) lower than those of CCF (89.48°C and 22.20 min). The lower pasting temperature of Formula 5 implies that starch gelatinization and paste formation can begin at a lower energy input compared to CCF, which may enhance its suitability for rapid cooking and energy‐efficient preparation, particularly important in resource‐constrained settings. Similarly, the significantly shorter peak time suggests faster thickening, which is beneficial for caregivers preparing complementary foods. In contrast, the higher gelatinization temperature and prolonged peak time in CCF may be indicative of more resistant starch structures, possibly due to stronger intermolecular bonding, higher protein or lipid interactions, or larger particle sizes, although granulometric analysis was not conducted in the present study to confirm this. The observed pasting behavior highlights the influence of ingredient composition and interaction in starch matrices. However, a direct comparison to other studies should be interpreted cautiously since variations in ingredient sources, processing methods, and particle size may significantly affect pasting properties.

**Table 6:** Functional and pasting properties of OFSP‐based complementary food.

	**Pasting properties**				
**Sample**	**Pasting temperature (°C)**	**Peak viscosity (cP)**	**Breakdown viscosity (cP)**	**Setback viscosity (BU)**	**Peak time (min)**
Formula 5 (60:30:10)	86.75 ± 0.07^a^	647 ± 1.41^a^	65 ± 0.00^a^	197 ± 1.41^a^	5.46 ± 0.02^a^
CCF	89.48 ± 0.80^b^	32.00 ± 2.64^b^	1.64 ± 0.38^b^	10.76 ± 0.22^b^	22.20 ± 0.53^b^
	**Functional properties**				
	**WAC (%)**	**Swelling power (g/g)**	**Solubility index (%)**	**Bulk density (g/mL)**	**OAC (%)**
Formula 5 (60:30:10)	140.95 ± 0.45^a^	9.25 ± 0.11^a^	14.01 ± 0.29^a^	0.64 ± 0.01^a^	115.89 ± 0.11^a^
CCF	25.00 ± 0.82^b^	11.25 ± 2.10^b^	7.32 ± 0.64^b^	0.73 ± 0.41^b^	23.36 ± 0.28^b^

*Note:* Sample ratios are represented as orange‐fleshed sweet potato:Bambara groundnut:brown rice. Mean values with different superscripts along the column are significantly different (*p* < 0.05).

Abbreviations: CCF, commercial complementary food; OAC, oil absorption capacity; WAC, water absorption capacity.

The peak viscosity, breakdown viscosity, and setback viscosity for Formula 5 were 647, 65 cP, and 197 (BU) relative to 32.00, 1.64 cP, and 0.76 BU of CCF (Table 6). Peak viscosity is the ability of starch to swell freely before its physical breakdown, and higher values indicate the food forms a highly viscous paste on cooking and cooling, whereas low values produce a paste of low viscosity [44]. The peak viscosity means Formula 5 forms a viscous paste during cooking and cooling. Breakdown viscosities reflect the stability of the peak viscosity during processing and show the extent of retrogradation, whereas the setback viscosity indicates gel stability and potential for retrogradation [45]. Higher breakdown and setback viscosities of Formula 5 indicate the starches are stable and can withstand high temperatures and also have a lower tendency to retrograde compared to CCF [45]. The physicochemical properties of OFSP, soybean, and groundnut reveal differences compared to other complementary products in terms of starch granule size, amylose content, and interaction with proteins and lipids, all of which significantly influence pasting behavior. Therefore, the higher viscosity observed in Formula 5 likely stems from the intrinsic compositional differences and ingredient synergy specific to this formulation.

#### 3.4.2. Functional Properties

Table 6 presents the functional properties of Formula 5 flour blend compared to CCF. The Formula 5 blend exhibited a WAC of 140.95%, a swelling power of 9.25 g/g, a solubility index of 14.01%, a bulk density of 0.64 g/mL, and an oil absorption capacity (OAC) of 115.89%. In contrast, CCF recorded a WAC of 25.00%, a swelling power of 11.25 g/g, a solubility index of 7.32%, a bulk density of 0.73 g/mL, and an OAC of 23.36%. WAC is a critical parameter that determines the ability of a flour to absorb and retain moisture, thus influencing its consistency in food formulations. The elevated WAC observed in Formula 5 suggests that it can hold moisture more effectively than CCF. The ability to hold water is vital for improving the texture, mouthfeel, and overall palatability of complementary foods for infants and young children [46]. The WAC property is strongly associated with the protein content, type of starch present, and ash content of the flour, as these factors influence water retention capacity [47]. Variations in the chemical composition [48], milling techniques [47], and the structural properties of the flour′s ingredients [48] significantly impact its WAC. According to McWatters et al. [49], differences in WAC are primarily linked to variations in protein concentrations. However, since the protein levels in Formula 5 (14.24%) and CCF (13.27%) are quite similar, the observed differences are attributed to the presence of hydrophobic amino acids in the protein, fat levels, mineral levels, and the starch′s conformational structure, which influence water absorption [50].

The OAC was highest in Formula 5 (115.89%) relative to CCF (23.36%). This difference can be linked to the biomolecular properties, including the fat and protein content of the flour [51]. A higher fat content generally promotes oil absorption due to the emulsifying properties of fat, which improve interactions between the flour matrix and oil [51]. However, despite having the highest fat content, CCF recorded the lowest OAC. This discrepancy is likely influenced by the amino acid composition of the protein and the starch granules of the carbohydrate. Proteins with higher levels of hydrophobic amino acids increase the flour′s hydrophobicity, enhancing its capacity to bind oil and, consequently, improving its oil absorption potential [50, 51]. Starch granules, depending on their size and structure, can physically entrap oil. Higher amylose content in starch influences its ability to form complexes with lipids, which can also enhance OAC [52].

Swelling power reflects the ability of starch granules to absorb water and expand, providing insight into the strength of the molecular bonds within the granule and the extent to which water interacts with the starch′s internal structure [53]. This characteristic is crucial for determining the texture and consistency of food products, particularly infant‐ and cereal‐based foods. Achieving the right consistency is essential for both sensory qualities and digestibility. Chandra et al. [54] emphasized that moderate to high swelling power is necessary to enhance the functional properties of flour in baby foods and breakfast cereals, ensuring smoothness and viscosity. In this study, the swelling power of Formula 5 (9.25 g/g) was significantly lower than that of CCF (11.25 g/g). The difference in swelling power can be attributed to the varying composition of the flours in the two samples, particularly the starch content as well as the arrangement and orientation of starch granules within the flour matrix, which affects water absorption and the ability of the flour to swell when hydrated [53].

Formula 5 demonstrated a higher solubility (14.01%) compared to CCF (7.32%). The increase in WAC enhances swelling power, which subsequently increases solubility [55]. This explains the greater solubility observed in Formula 5 compared to CCF. The solubility of flour provides insight into how well the particles dissolve in a given solvent, which plays a significant role in determining its applications [55]. A higher solubility is often linked to a greater degree of starch gelatinization, which affects the flour′s ability to be incorporated into various food systems. This is particularly important in the context of infant formula and complementary foods, as higher solubility generally correlates with improved digestibility. As a result, foods with a higher solubility index are often more suitable for infants and young children, where ease of digestion is crucial [55].

The bulk density of Formula 5 (0.64 g/mL) was lower compared to CCF (0.73 g/mL). The difference in bulk density is influenced largely by the structural arrangement of starch polymers within the sample. A looser packing of starch molecules results in a lower bulk density, while a tighter structure leads to a higher bulk density [43]. This characteristic not only reflects the material′s interaction in dry mixes but also has significant implications for packaging and storage requirements, as products with lower bulk density require larger packaging volumes [56]. The bulk density of Formula 5, measured at 0.64 g/mL, suggests a relatively loose starch structure compared to that of CCF. This indicates that only a small volume of water is needed to prepare a larger mass of the formula, making it easier for infants to consume without risking choking or discomfort [47]. This is a key advantage of complementary foods, as it facilitates feeding, particularly in infants who are still developing swallowing coordination.

## 4. Conclusion

The most preferred formulation of the OFSP‐based complementary food, consisting of 60% OFSP, 30% Bambara groundnut, and 10% brown rice flour, demonstrated superior nutritional and functional qualities compared to the CCF. This blend exhibited significantly higher levels of protein (14.24%), fiber (4.50%), ash (2.77%), carbohydrate (73.78%), and *β*‐carotene (541.4 mg/100 g), providing enhanced nutritional value for infants and young children. The most preferred formulation also contained substantially higher concentrations of key minerals, including Ca (185.3 mg/100 g), Fe (5.84 mg/100 g), K (261.33 mg/100 g), and Mg (204.75 mg/100 g). However, its Zn content (1.20 mg/100 g) was lower than CCF′s. Moreover, this formulation met the nutritional recommendations established by the FAO and CAC for children aged 6–23 months, highlighting its potential to serve as an effective, nutrient‐dense complementary food. The use of OFSP flour, in combination with Bambara groundnut and brown rice, offers a promising alternative for households with limited resources, particularly in regions where nutrient deficiencies are prevalent. Furthermore, the blend′s optimal functional and pasting properties ensured a desirable viscosity when prepared as a porridge, enhancing its suitability for infant feeding. This study underscores the potential of locally sourced ingredients, such as OFSP, Bambara groundnut, or brown rice, to contribute to the development of nutritionally balanced, affordable, and culturally appropriate complementary foods, particularly in resource‐limited settings.

## Ethics Statement

The protocol for the study was reviewed and approved by the Committee on Human Research, Publications and Ethics of the School of Medical Sciences, Kwame Nkrumah University of Science and Technology (Approval Number: CHRPE/AP/178/23), Kumasi, Ghana, before the commencement of the activities of the study. Participation in the study was voluntary, and participants who agreed to participate in the study were made to thumbprint or append their signatures on a participant consent form in the presence of a witness before participating in sensory analysis.

## Conflicts of Interest

The authors declare no conflicts of interest.

## Author Contributions

**Doreen Ehornam Alomatu:** conceptualization, formal analysis, investigation, resources, methodology, writing – review and editing. **Shadrach Yankey:** conceptualization, formal analysis, investigation, methodology, project administration, supervision, data curation, visualization, writing – original draft, writing – review and editing. **Linda Gyimah:** conceptualization, supervision, methodology, writing – review and editing. **Guy Eshun:** conceptualization, supervision, methodology, writing – review and editing.

## Funding

No funding was received for this manuscript.

## Data Availability

All relevant data are within the manuscript.

## References

[1] Ojinnaka M. C. , Ebinyasi C. S. , Ihemeje A. , and Okorie S. U. , Nutritional Evaluation of Complementary Food Gruels Formulated From Blends of Soybean Flour and Ginger Modified Cocoyam Starch, Advance Journal of Food Science and Technology. (2013) 5, no. 10, 1325–1330, 10.19026/ajfst.5.3105, 2-s2.0-84887556023.

[2] Amagloh F. K. , Weber J. L. , Brough L. , Hardacre A. , Mutukumira A. N. , and Coad J. , Complementary Food Blends and Malnutrition Among Infants in Ghana: A Review and a Proposed Solution, Scientific Research Essays. (2012) 7, 972–988, 10.5897/SRE11.1362.

[3] Tengepare F. , Improving Maternal and Child Nutrition Services in Community Based Health Planning and Services Zones in the Jirapa Municipality of Northern Ghana- Challenges and Strategies: The Perspective of Community Health Officers, BMC Nutrition. (2023) 10, 10.1186/s40795-024-00848-8. PMC11179358 38877539

[4] Greffeuille V. , Dass M. , Fanou-Fogny N. , Nyako J. , Berger J. , and Wieringa F. , Micronutrient Intake of Children in Ghana and Benin: Estimated Contribution of Diet and Nutrition Programs, Maternal and Child Nutrition. (2022) 19, no. 2, 10.1111/mcn.13453. PMC10019049 36394283

[5] Laryea D. , Wireko-manu F. D. , and Oduro I. , Formulation and Characterization of Sweetpotato- Based Complementary Food, Cogent Food & Agriculture. (2018) 4, no. 1, 1–15, 10.1080/23311932.2018.1517426.

[6] Low J. W. , Mwanga R. O. M. , Andrade M. , Carey E. , and Ball A. M. , Tackling Vitamin A Deficiency With Biofortified Sweetpotato in Sub-Saharan Africa, Global Food Security. (2017) 14, 23–30, 10.1016/j.gfs.2017.01.004, 2-s2.0-85015287933. 28989861 PMC5614018

[7] Mohammed A. , Shiferaw G. , and Bajigo A. , The Effect of Maize-Cowpea Blends on Complementary Food Nutritional Quality, Global Journal of Science Frontier Research: I Interdisciplinary. (2016) 16, no. 2, 1–11.

[8] Asravor J. , Wiredu A. N. , Siddig K. , and Onumah E. E. , Evaluating the Environmental-Technology Gaps of Rice Farms in Distinct Agro-Ecological Zones of Ghana, Sustainability. (2019) 11, no. 7, 10.3390/su11072072, 2-s2.0-85064085700.

[9] Atoyebi J. O. , Osilesi O. , Adebawo O. O. , and Abberton M. , Evaluation of Nutrient Parameters of Selected African Accessions of Bambara Groundnut (Vigna subterranea (L.) Verdc.). *American* , Journal of Food and Nutrition. (2017) 5, no. 3, 83–89, 10.12691/AJFN-5-3-1.

[10] Chinedu S. N. and Nwinyi C. O. , Proximate Analysis of Sphenostylis stenocarpa and Voadzeia subterranean Consumed in South Eastern Nigeria, Journal of Agricultural Extension and Rural Development. (2012) 4, no. 3, 57–62, 10.5897/JAERD11.031.

[11] Nwadi O. , Uchegbu N. , and Oyeyinka S. , Enrichment of Food Blends With Bambara Groundnut Flour: Past, Present, and Future Trends, Legume Science. (2020) 2, no. 1, e25, 10.1002/leg3.25.

[12] Alomatu D. , Yankey S. , Gyimah L. , and Eshun G. , Nutritional Profile and Functional Properties of Orange Flesh Sweet Potato, Bambara Groundnut, and Brown Rice-Base Complementary Food, https://ssrn.com/abstract=4988570. 10.1155/ijfo/5397972 PMC12519420 41098858

[13] Marcel R. , Mary J. , and S., Chacha, C., & Ofoedu, E. , Nutritional Evaluation of Complementary Porridge Formulated From Orange Fleshed Sweet Potato, Amaranth Grain, Pumkin Seed, and Soybean Flours, Food and Nutrition. (2021) 10, no. 4, 536–553, 10.1002/fsn3.2675. PMC8825733 35154690

[14] Alawode E. K. , Idowu M. A. , Adeola A. A. , Oke E. K. , and Omoniyi S. A. , Some Quality Attributes of Complementary Food Produced From Flour Blends of Orange Flesh Sweetpotato, Sorghum, and Soybean, Croatian Journal of Food Science and Technology. (2017) 9, no. 2, 122–129, 10.17508/CJFST.2017.9.2.06.

[15] AOAC , Official Methods of Analysis, 2005, 18th edition, Association of Official Analytical Chemists.

[16] CAC , 2011, Guidelines on Formulated Supplementary Foods for Older Infants and Young Children, Proposed Draft Revision, Codex Alimentarius Commission, Joint FAO/WHO Food Standards Programme, Codex Committee on Nutrition and Foods For Special Dietary Uses, CX/NFSDU 11/33/8.

[17] Munasinghe M. and Wansapala J. , *β*-Carotene Content of M. longifolia Seed Oil in Different Agroclimatic Zones in Sri Lanka, the Effect of Heat on Its Stability and the Composition of Seed Cake, Potravinarstvo. (2015) 9, no. 1, 474–479, 10.5219/502, 2-s2.0-84963525785.

[18] Effah-Manu L. , Wireko-Manu F. D. , Agbenorhevi J. K. , Maziya-Dixon B. , and Oduro I. , Chemical, Functional and Pasting Properties of Starches and Flours From New Yam Compared to Local Varieties, CYTA–Journal of Food. (2022) 20, no. 1, 120–127, 10.1080/19476337.2022.2093401.

[19] Nchung L. , Eke M. O. , Emmanuel C. N. , and Bongjo N. B. , Assessment of Nutritional and Functional Properties of Complementary Food from Orange-Fleshed Sweet Potato, Soybean and Tropical Almond Seed Composite Flour, European Journal of Nutrition & Food Safety. (2024) 16, no. 2, 10.9734/ejnfs/2024/v16i21384.

[20] Sebben J. A. , Trierweiler L. F. , and Trierweiler J. O. , Orange-Fleshed Sweet Potato Flour Obtained by Drying in Microwave and Hot Air, Journal of Food Processing and Preservation. (2016) 41, no. 1, e12744, 10.1111/jfpp.12744, 2-s2.0-84964350395.

[21] Etana G. , Belew D. , and Beyene T. M. , Proximate Composition of Orange Fleshed Sweet Potato (*Ipomoea batatas* (L.) *Lam*) Varieties as Influenced by Blended Fertilizer Level, AGBIR. (2022) 38, no. 1, 252–258.

[22] Linnemann A. R. , Bambara Groundnut (*Vigna subterranea* (L.) Verdc.): A review, Abstracts on Tropical Agriculture. (1987) 12, no. 7, 9–25.

[23] Hillocks R. J. , Bennett C. , and Mponda O. M. , Bambara Nut: A Review of Utilisation, Market Potential and Crop Improvement, African Crop Science Journal. (2012) 20, no. 1, 1–16, 10.4314/acsj.v20i1.

[24] Mazahib A. M. , Uha M. O. , Salawa I. , and Babiker E. E. , Some Nutritional Attributes of Bambara Groundnut as Influenced by Domestic Processing, International Food Research Journal. (2013) 20, no. 3, 1165–1171.

[25] Olapade A. and Adetuyi D. O. , Comparison of Different Methods of Producing Bambara (*Voandzeia subterranean* L. Thou) Flours for Preparation of ‘Moin-Moin’, Nigerian Food Journal. (2007) 25, no. 2, 150–157, 10.4314/nifoj.v25i2.50853.

[26] Wireko-manu F. D. and Amamoo C. , Comparative Studies on Proximate and Some Mineral Composition of Selected Local Rice Varieties and Imported Rice Brands in Ghana, Agriculture and Food Sciences Research. (2017) 4, no. 1, 1–7, 10.20448/journal.512.2017.41.1.7.

[27] Honi B. , Mukisa I. M. , and Mongi R. J. , Proximate Composition, Provitamin A Retention, and Shelf Life of Extruded Orange-Fleshed Sweet Potato and Bambara Groundnut-Based Snacks, Journal of Food Processing and Preservation. (2017) 2, no. 1, e13415, 10.1111/jfpp.13415, 2-s2.0-85023638413.

[28] Rezende L. V. , Paraizo W. B. , Santos J. D. A. , Amorim K. A. , Goulart G. A. S. , Becker F. S. , and Damiani C. , Study of the Drying Process of the Purple-Fleshed Sweet Potato (*Ipomoea batatas* (L.) Lam) in Spouted Bed, Food Science and Technology. (2024) 44, e00180.

[29] FAO. (1990). Roots, tubers, plantains and bananas in human nutrition (FAO Food and Nutrition Series No. 24). Food and Agriculture Organization of the United Nations. https://www.fao.org/4/t0207e/t0207e01.htm#:%7E:text=David%20Lubin%20Memorial%20Library%20Cataloguing,24, ). 6086142

[30] CAC (1991). Guidelines on formulated complementary foods for older infants and young children (CAC/GL 8-1991). FAO/WHO Joint Publications. Food and Agriculture Organization of the United Nations. https://www.fao.org/fao-who-codexalimentarius/sh-proxy/en/?lnk=1%26url=https%253A%252F%252Fworkspace.fao.org%252Fsites%252Fcodex%252FStandards%252FCXG%2B8-1991%252FCXG_008e.pdf.

[31] Bonsi E. A. , Plahar W. A. , and Zabawa R. , Nutritional Enhancement of Ghanaian Weaning Foods Using the Orange Flesh Sweetpotato (*Ipomea batatas*), African Journal of Food Agriculture Nutrition Development. (2014) 14, no. 65, 9236–9256, 10.18697/ajfand.65.13190.

[32] Adenuga W. , Nutritional and Sensory Profiles of Sweet Potato Based Infant Weaning Food Fortified With Cowpea and Peanut, Journal of Food Technology. (2010) 8, no. 5, 223–228, 10.3923/jftech.2010.223.228.

[33] FAO/WHO/UNICEF , Protein Advisory Group: Guideline for Human Testing of Supplementary Food Mixtures, 1972, United Nations.

[34] Adigwe N. E. , Kiin-Kabari D. B. , and Emelike N. J. T. , Nutritional Quality and In Vitro Protein Digestibility of Complementary Foods Formulated From Maize, Cowpea and Orange-Fleshed Sweet Potato Flours: A Preliminary Study, Asian Food Science Journal. (2023) 22, no. 2, 25–37, 10.9734/afsj/2023/v22i2619.

[35] Haque M. R. , Hosain M. M. , Khatun H. , Alam R. , and Gani M. O. , Evaluation of Nutritional Composition and Sensory Attributes of Weaning Food Prepared From Sweetpotato and Soyabean, Bangladesh Research Publications Journal. (2013) 8, no. 2, 127–133.

[36] Adedayo O. E. , Yisa O. O. , Olanrewaju O. I. , and Dele-Olawumi B. , Nutrients and Antioxidants Composition of Complementary Foods Produced From Brown Local Rice, Soybean, and Tiger Nut Supplemented With Orange-Fleshed Sweet Potato, Journal of Dietitians Association of Nigeria. (2022) 12, no. 1, 61–71, 10.4314/jdan.v12i1.9.

[37] Oko A. O. , Ubi B. E. , Efisue A. A. , and Dambaba N. , Comparative Analysis of the Chemical Nutrient Composition of Selected Local and Newly Introduced Rice Varieties Grown in Ebonyi State of Nigeria, International Journal of Agriculture and Forestry. (2012) 2, no. 2, 16–23, 10.5923/j.ijaf.20120202.04.

[38] Koletzko B. , Cooper P. , Makrides M. , Garza C. , Uauy R. , and Wang W. , Pediatric Nutrition in Practice, 2008, Karger Medical and Scientific Publishers.

[39] Booth S. L. , Lichtenstein A. H. , O′Brien-Morse M. , McKeown N. M. , Wood R. J. , Saltzman E. , and Gundberg C. M. , Effects of a Hydrogenated Form of Vitamin K on Bone Formation and Resorption, American Journal of Clinical Nutrittion. (2001) 74, 783–790, 10.1093/ajcn/74.6.783. 11722960

[40] Martínez-Ballesta M. C. , Domínguez-Perles R. , Moreno D. A. , Muries B. , Alcaraz-López C. , Bastías E. , García-Viguera C. , and Carvajal M. , Lichtfouse E. , Hamelin M. , Navarrete M. , and Debaeke P. , Minerals in Plant Food: Effect of Agricultural Practices and Role in Human Health, Sustainable Agriculture, 2011, Springer, 111–128, 10.1007/978-94-007-0394-0_8, 2-s2.0-84886236996.

[41] Younge S. , Amoah R. , Abano E. , Kumi F. , and Anyebuno G. , Physico-Nutritional Characterization of Composite Cassava and Orange-Fleshed Sweet Potato Flours and Sensory Evaluation of “Fufu” Prepared from the Flour Blends, Journal of Food Processing and Preservation. (2022) 46, no. 12, e17254, 10.1111/jfpp.17254.

[42] Di Bella G. , Naccari C. , Bua G. D. , Rastrelli L. , Lo Turco V. , Potortì A. G. , and Dugo G. , Mineral Composition of Some Varieties of Beans From Mediterranean and Tropical Areas, International Journal of Food Sciences and Nutrition. (2016) 67, no. 3, 239–248, 10.3109/09637486.2016.1153610, 2-s2.0-84961205928, 26940501. 26940501

[43] Eke-Ejiofor J. , Obinna-Echem P. C. , Wordu G. O. , and Vito M. B. , Physicochemical, Functional and Pasting properties of Orange-Flesh Sweet Potato Starch, Soya Bean and Groundnut Flour Complementary Food, American Journal of Food Science and Technology. (2021) 9, no. 3, 96–104, 10.12691/ajfst-9-3-5.

[44] Ikujenola A. V. and Fashakin J. B. , The Physicochemical Properties of a Complimentary Diet Prepared From Vegetable Protein, Journal of Food Agriculture and Environment Finland. (2005) 3, 23–26.

[45] Babajide J. M. and Olowe S. , Chemical, Functional and Sensory Properties of Water Yam – Cassava Flour and Its Paste, International Food Research Journal. (2013) 20, no. 2, 903–909.

[46] Badiora O. , Some Quality Properties of Yellow-Fleshed Sweet Potato Flour as Affected by Different Drying Methods, Food Production Processing and Nutrition. (2023) 5, no. 1, 10.1186/s43014-023-00136-1.

[47] Anosike F. C. , Nwagu K. E. , Nwalo N. F. , Ikegwu O. J. , Onyeji G. N. , Enwere E. N. , and Nwoba S. T. , Functional and Pasting Properties of Fortified Complementary Foods Formulated From Maize (*Zea mays*) and African Yam Bean (*Sphenostylis stenocarpa*) Flours, Legume Science. (2020) 2, no. 4, 1–11, 10.1002/leg3.62.

[48] Zhang J. , Tao L. , Yang S. , Ye L. , Qi W. , Shixin S. , and Lei Y. , Water Absorption Behavior of Starch: A Review of Its Determination Methods, Influencing Factors, Directional Modification, and Food Applications, Trends in Food Science & Technology. (2023) 144, 104321, 10.1016/j.tifs.2023.104321.

[49] McWatters K. H. , Ouedraogo J. B. , Resurreccion A. V. A. , Hung Y. , and Phillips R. D. , Physical and Sensory Characteristics of Sugar Cookies Containing Mixtures of Wheat, Fonio (*Digitaria exilis*) and Cowpea (*Vigna unguiculata*) Flours, International Journal of Food Science and Technology. (2003) 38, 403–410, 10.1046/j.1365-2621.2003.00716.

[50] Türker D. A. , Determination of the Physical Quality, Structural Characteristics, and Sensory Acceptability of Biscuits Prepared From Einkorn Based Lentil Composite Flours, Harran Journal of Agricultural and Food Science. (2024) 28, no. 3, 430–443, 10.29050/harranziraat.1477200.

[51] Kumari B. , Physicochemical, Rheological Properties, and In-Vitro Starch Digestibility of Flours and Starches From Pigeon Pea, Cowpea, Pinto Bean, and Navy Bean, Starch-Stärke. (2024) 76, 9–10, 10.1002/star.202300303.

[52] Li L. , Chen J. , Bai D. , Xu M. , Cao W. , Ren G. , Ren A. , and Duan X. , Physicochemical, Pasting Properties and In Vitro Starch Digestion of Chinese Yam Flours as Affected by Microwave Freeze-Drying, Food. (2022) 11, no. 15, 10.3390/foods11152324. PMC9368656 35954090

[53] Li M. , Zhang Y. , You X. , Wang Y. , Zhu K. , Wei P. , and Wei L. , Assessment of Functional Properties of Wheat–Cassava Composite Flour, Food. (2023) 12, no. 19, 10.3390/foods12193585, 37835238. PMC10572405 37835238

[54] Chandra S. , Singh S. , and Kumari D. , Evaluation of Functional Properties of Composite Flours and Sensorial Attributes of Composite Flour Biscuits, Journal of Food Science and Technology. (2015) 52, no. 6, 3681–3688, 10.1007/s13197-014-1427-2, 2-s2.0-84929965311. 26028751 PMC4444897

[55] Oppong D. , Arthur E. , Osei K. , Badu E. , and Sakyi P. , Proximate Composition and Some Functional Properties of Soft Wheat Flour, International Journal of Innovative Research in Science, Engineering and Technology. (2015) 4, no. 2, 753–758, 10.15680/IJRSET.2015.0402097.

[56] Mohamed T. K. , Zhu K. , Issoufou A. , Fatmata T. , and Zhou H. , Functionality, *In Vitro* Digestibility and Physicochemical Properties of Two Varieties of Defatted Foxtail Millet Protein Concentrates, International Journal of Molecular Sciences. (2009) 10, 5224–5238, 10.3390/ijms10125224, 2-s2.0-74249107293.

[57] Mahmoud A. and El-Anany A. , Nutritional and Sensory Evaluation of a Complementary Food Formulated From Rice, Faba Beans, Sweet Potato Flour, and Peanut Oil, Food and Nutrition Bulletin. (2014) 35, no. 4, 403–413, 10.1177/156482651403500402, 2-s2.0-84925545493. 25639125

